# A Novel Machine Learning Model for the Automated Diagnosis of Nasal Pathology in Canine Patients

**DOI:** 10.3390/ani15121718

**Published:** 2025-06-10

**Authors:** Andreea Istrate, Radu Constantinescu, Lithicka Anandavel, Shraddha Rajeshkumar Tandel, Simon Dye, Charlotte Dye

**Affiliations:** 1 Pride Veterinary Referrals, Independent Vetcare (IVC) Evidensia, Riverside Road, Derby DE24 8HX, UK; c.dye@live.co.uk; 2 Dick White Referrals Veterinary Specialists, Part of Linnaeus, Station Farm, London Road, Six Mile Bottom, Cambridgeshire CB8 0UH, UK; constantinescur.radu@gmail.com; 3 School of Physics and Astronomy, University of Nottingham, University Park, Nottingham NG7 2RD, UK; lithicka@gmail.com (L.A.); shraddhatandel1106@gmail.com (S.R.T.); simon.dye@nottingham.ac.uk (S.D.)

**Keywords:** convolutional neural network, machine learning, computed tomography, intranasal neoplasia, fungal rhinitis, canine head, veterinary medicine

## Abstract

Computed tomography (CT) is the imaging method of choice for evaluating the canine nasal cavity, being invaluable in determining disease extent, guiding sampling, and planning treatment. While predictions of pathology type can be made, there is significant overlap between CT changes noted in neoplastic, inflammatory, and infectious nasal disease. Recent years have seen remarkable advancement in computer-aided detection systems in human medicine, with machine and deep learning techniques being successfully applied for the identification and accurate classification of intranasal pathology. This study aimed to develop a neural network pipeline for differentiating nasal pathology in dogs using CT studies of the head. A total of 80 CT studies were recruited for training and testing purposes. Studies falling into one of the three groups (normal nasal anatomy, fungal rhinitis, and intranasal neoplasia) were manually segmented and used to train a suite of neural networks. Standard accuracy metrics assessed performance during training and testing. The machine learning algorithm showed reasonable accuracy (86%) in classifying the diagnosis from an isolated scan slice but high accuracy (99%) when aggregating over slices taken from a full scan. These results suggest that machine learning programmes can accurately discriminate between intranasal pathologies based on canine computed tomography.

## Simple Summary

Computed tomography (CT) is the modality of choice for assessing the canine nasal cavity, offering critical insights into disease extent, facilitating targeted tissue sampling, and informing therapeutic strategies. Although CT findings can provide indications of pathology type, considerable overlap exists among neoplastic, inflammatory, and infectious nasal diseases, complicating definitive differentiation. In human medicine, recent advancements in computer-aided detection have leveraged machine learning and deep learning techniques to enhance the identification and classification of intranasal pathology with high accuracy. This study aimed to develop a neural network-based pipeline for the automated detection and classification of nasal pathology in canines using CT imaging. A dataset comprising 80 CT studies of the head was curated for model training and validation. Each study was assigned to one of three categories—normal nasal anatomy, fungal rhinitis, or intranasal neoplasia—and manually segmented to train a series of neural networks. Performance was evaluated using standard accuracy metrics. The trained model demonstrated a classification accuracy of 86% on isolated image slices and a diagnosis accuracy of 99% when aggregated across slices of a given patient. These findings underscore the potential of machine learning algorithms in accurately differentiating intranasal pathologies in canines, highlighting their applicability in augmenting diagnostic workflows and advancing veterinary imaging.

## 1. Introduction

The use of machine learning is a rapidly growing field in medical imaging and can achieve high degrees of accuracy that can exceed the abilities of human judgement in making data predictions [1]. It has numerous clinically relevant uses and, in human medicine, is already being used to aid in disease detection, diagnosis, prognosis, and treatment response [2]. While little information is currently available regarding the use of machine learning in veterinary species, there are clear areas of utility and an expectation that it will have a significant impact with regard to medical decision making in the future.

The concept of using machine learning to assess nasal and paranasal pathology is well described in human medicine, with multiple publications describing its utility for the detection and classification of various pathologies, including rhinosinusitis [3,4,5,6], nasal polyps [7,8], fungal infections [9], and malignancies [10,11,12,13]. Given the much wider inter-species and inter-breed anatomical variation in veterinary species, proof of concept and pilot studies are required to confirm that similar machine learning techniques can be applied; however, the initial results are promising [14]. To the author’s knowledge, there are only two publications specifically describing the application of machine learning to images of the canine head and neck, which described a highly consistent and robust model for the delineation of radiotherapy planning [15,16].

There is an overlap between the CT features of inflammatory, infectious, and neoplastic nasal pathologies, with varying degrees of nasal turbinate destruction, soft tissue-attenuating material within the nasal cavities and/or frontal sinuses, and cribriform plate lysis being just a few of the common characteristics [17,18,19]. In particular, both fungal and neoplastic diseases often exhibit invasive and infiltrative behaviours within the nasal cavities [20,21]. Although there are several distinguishing features that can help determine the diagnosis, further investigations, such as rhinoscopy and biopsies, are usually required to reach an accurate diagnosis [21]. Definitive diagnosis therefore relies on histopathology, but the acquisition of representative tissue biopsies is invasive and lesions are not always easily accessible [22,23]. The ability of machine learning to use raw rather than reconstructed data offers an opportunity for increased sensitivity and specificity in comparison with human interpretation, raising the possibility of yielding enhanced diagnostic and prognostic information and perhaps reducing the need for histopathologic confirmation in the future [2].

Machine learning is an umbrella term that refers to a broad range of algorithms that perform intelligent predictions based on a dataset. A wide variety of models are in use, the choice of which is determined by the characteristics of the data and the type of desired outcome. In the case of image classification tasks, the data lends itself to ‘supervised’ teaching models using input data that has predefined output labels associated with it [24]. The aim of this pilot study was to ascertain whether ‘supervised’ machine learning models could be used to identify the presence or absence of nasal pathology in dogs and to assess whether algorithms were able to further distinguish between normal nasal anatomy, fungal rhinitis, and intranasal neoplasia.

## 2. Materials and Methods

### 2.1. Selection and Categorisation of Subjects

CT data from two large UK veterinary referral hospitals, stored via two Picture Archiving and Communication Systems (PACS), Horos (Horos version 3.3.6, The Horos Project, Pureview) and Osirix (Osirix MD DICOM viewer Viewer, Pixmeo Sarl, version 14.0.1, Geneva, Switzerland), between the dates of 2013 and 2022, were interrogated for all canine anatomical studies labelled ‘head CT’. All studies were obtained using a multidetector row scanner (BrightSpeed [16 slice], General Electric Medical Systems, Chicago, IL, USA or MX 8000 IDT, Philips Medical Systems, Cleveland, OH, USA [16 slice]), depending on the hospital of origin. The studies were visually assessed by two of the authors (A.I. and R.C), and those of diagnostic quality from dogs with and without nasal pathology were archived. A final diagnosis along with patient signalment for each of the archived studies was then documented based on information derived from specialist CT reports, clinical notes, and laboratory reports. Ethical approval was granted by the Royal College of Veterinary Surgeons Ethics Review Pannel (2022-108).

CT studies were initially separated into two groups (normal or abnormal nasal anatomy), based on the presence or absence of nasal pathology according to the findings documented in the CT reports at the time of diagnosis. All included CT studies were reported by Diplomates in Veterinary Diagnostic Imaging (ECVDI). A definitive diagnosis of either fungal rhinitis (*Aspergillus fumigatus* infection) or intranasal neoplasia (lymphoma, carcinoma or sarcoma) was then assigned to each study in the ‘abnormal’ group based on the clinical notes and laboratory results. Studies were excluded if a definitive histopathologic diagnosis was not available. A total of 80 CT studies were selected for model training: 27 with ‘normal nasal anatomy’, 28 with ‘fungal rhinitis’, and 25 in the ‘neoplasia’ subgroup. All studies were acquired from medium- to large-headed dolichocephalic breeds.

### 2.2. Data Preparation

Due to the examinations spanning almost a decade, CT scanning protocols and parameters were variable between institutions. Nevertheless, all scans included pre- and post-contrast imaging with soft-tissue and bone algorithms. Based on preliminary investigations, the more identifiable anatomical structures and extent of pathology demonstrated in the bone pre-contrast scans allowed for more efficient and reliable training of the classification model; hence, only the bone pre-contrast window images were utilised for the remainder of the study.

CT data were exported as Digital Imaging and Communications in Medicine (DICOM) files. For training the slice selection and segmentation models, volumetric masks encompassing the nasal cavities in all 80 patients were manually defined by two of the authors (L.A. and S.T.) using the Medical Image Labeller within MATLAB (Version: 9.13.0, R2022b). DICOM scan files and masks were then converted to Neuroimaging Informatics Technology Initiative (NIfTI) format using the python library dicom2nifti (Version: 2.4.8), to bring all scan and mask data to a common resolution for model training (see below). All analysis in the study was restricted to scan data projected onto the axial plane. Aggregating all 80 patients, a total number of approximately 12,000 axial slices intersecting the nasal cavity were acquired. Section 2.5 outlines how slices were split into training, validation, and testing sets.

When converting to NIfTI format, a set of scans retaining the full image extent were created alongside a set of cropped scans. The cropped scans were formed by cropping each slice to a square bounding box containing all image pixels above a defined brightness threshold.

**Figure 1:**
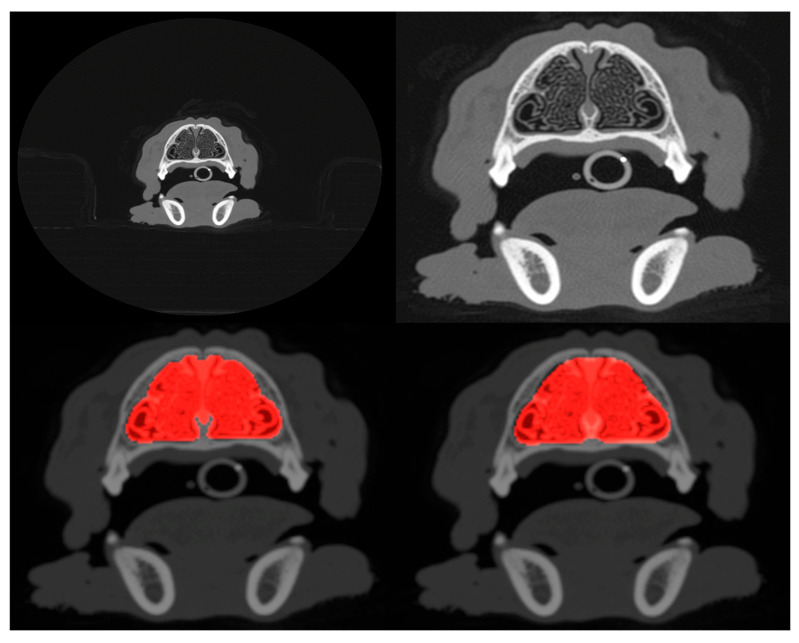
Example of uncropped (**top-left**) and cropped (**top-right**) scans projected onto the axial plane. Both cropped and uncropped images were tested in slice selection and segmentation, but only cropped images were used for pathology classification. The **bottom-left** panel shows the manually defined ground truth mask (highlighted in red) that segments the nasal cavity of the slice shown in the **top-right**, and the **bottom-right** panel shows the mask predicted by the segmentation model.

Figure 1 shows an example of the extent of cropping. Both cropped and uncropped slices were created with image dimensions of 96 × 96 pixels and 128 × 128 pixels, reduced from the original dimensions of 512 × 512 pixels of the DICOM scans. Models were trained and tested with different combinations of image resolution and cropping (see next Section 2.3).

### 2.3. Models

A three-phase pipeline was assembled to evaluate the feasibility of fully automating nasal pathology diagnosis from CT scans. Each phase incorporated a different deep learning model. All models constructed by the authors utilise the tensorflow python library (Version: 2.9.0).

The first phase employs a model to select the range of axial slices that exclusively contains the nasal cavities from the most rostral region to the area of the cribriform plate. Several different model architectures were trained and tested on both cropped and uncropped 96 × 96-pixel slices. Models included very deep networks such as the *VGG16* convolutional neural network (CNN) [25], *ResNet50V2* [26], and *MobileNetV2* [27], but a shallow CNN constructed by the authors outperformed the deeper models. This shallow CNN comprises three convolutional blocks (64, 128, 256 filters, 3 × 3 kernel, each with 2,2 stride and each followed by 2 × 2 max-pooling) and three ReLU-activated dense layers (comprising 158, 256 and 128 neurons), with a final 1-channel sigmoid output. Given the shallow CNN’s superior performance, only its results are presented.

The second phase segments the nasal cavity regions within the slices selected in phase 1. A range of models based on the UNet architecture [28] were trained and tested on both cropped and uncropped 96 × 96-pixel slices. Of all the models tested, *ResUNet++* [29] showed the best performance; thus, only results from this model were reported. In this work, the network’s design preserves the native *ResUNet++* architecture. The encoder segment commences with a 16-filter input ResNet block, then progresses through three subsequent ResNet blocks containing 32, 64, and 128 filters, respectively. This progression extends to the bridge, which is characterised by 256 filters.

In the third and final phase, segmented regions are fed to a classification model. For this phase, two models were trained and tested on the 96 × 96- and 128 × 128-pixel cropped slices. The first model is a CNN constructed by the authors. Its architecture deepens through three convolutional blocks (comprising 32, 64, and 128 filters with 3 × 3 kernels, each followed by 2 × 2 max-pooling). The pooled features are then processed by a global average pooling layer, followed by a 512-neuron dense layer that uses ReLU activation and a 0.5 dropout rate. Finally, a softmax output layer provides the three classification probabilities. For the second, the 20-layer Residual Attention Network, *ResAttNet* [30], was used. The 3-channel implementation of the network applied in this study used a regularisation strength of 0.01 and a kernel size of 5 × 5 pixels in the convolutional layer.

### 2.4. Metrics

Several metrics were used to assess model performance during training and testing. For slice selection, models were trained using the binary cross-entropy loss function and tested using the standard metrics of accuracy (defined as the fraction of correct classifications out of all classifications made), the area under the Receiver Operating Characteristic curve (ROC-AUC), and the F1 score:

$$F1=\frac{2\times \mathrm{precision}\times \mathrm{recall}}{\mathrm{precision}+\mathrm{recall}}$$

where precision is the fraction of true positive classifications out of all positive classifications made, and recall is the fraction of true positive classifications out of all true cases. The F1 score provides a balanced metric even when a class imbalance exists. For segmentation, the testing metrics reported are the Dice coefficient, which measures the ratio of intersection to union between the true and predicted segmented area, and the F1 score. In the case of segmentation, for the F1 score, the precision measures the percentage of pixels predicted as positive that match the ground truth positive pixels. Recall is the percentage of actual positive pixels in the ground truth that are correctly identified by the model as positive. For training, the following definition of Dice loss was used: dice loss = 1 − dice coefficient. For the classification model, accuracy, the F1 score, and the ROC-AUC (in this case the ‘one versus rest’ strategy) were used as testing metrics. The cross-entropy loss function was used for training.

### 2.5. Model Training

In total, 20 instances of each model were trained; four unique sets of 20 patients from the cohort of 80 were used as unseen training data, and for each of these sets, 5 model instances were trained using 5-fold cross-validation with a 48/12 patient train/validation split. This helps mitigate the impact of the small patient cohort and enables the estimation of metric uncertainty through variance. Care was taken to ensure that the same approximately equal distribution of classes was present in the training and validation splits.

During training, all models utilised the ADAM optimizer. The slice extraction model was trained with a learning rate of 0.001 and a batch size of 256 slices and the segmentation model with a learning rate of 0.001 and batches of 64 slices. For the two classification models, the CNN was trained with a learning rate of 0.008 and batches of 256 slices and the *ResAttNet* model with a learning rate of 0.001 and a batch size of 32 slices. 

For all model training, early stopping was applied with a patience of 10 epochs. This prevents overfitting by monitoring the model’s performance on the validation dataset and stopping the training process when the loss stops improving for 10 epochs. Test and validation loss curves were manually monitored to confirm the absence of overfitting.

## 3. Results

**Figure 2:**
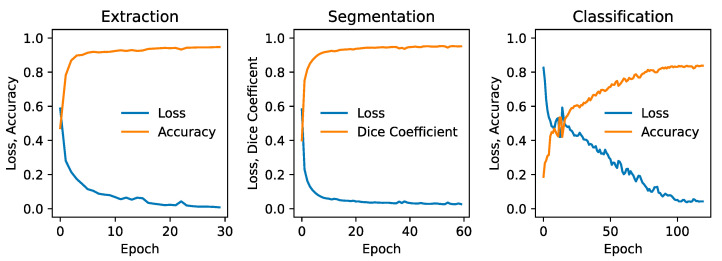
Validation metrics for each phase of the pipeline, showing the loss and accuracy for the slice extraction model (**left**), the loss and Dice coefficient for the segmentation model (**middle**), and the loss and accuracy for the slice classification CNN model (**right**). All metrics correspond to 96 × 96-pixel cropped slices.

**Figure 3:**
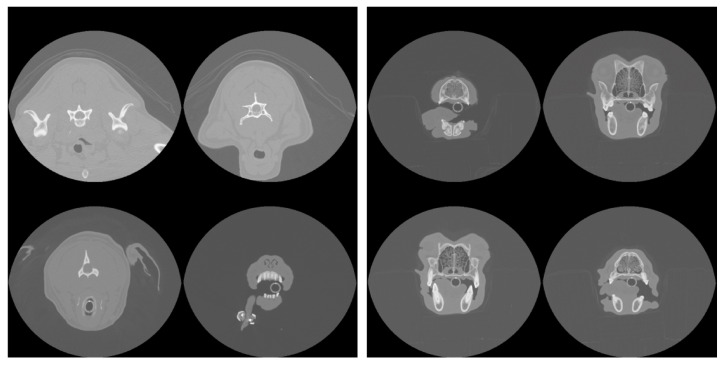
Example slices rejected (**left**) and accepted (**right**) by the model as intersecting the nasal cavity in one patient.

The metrics corresponding to the CNN model applied to both cropped and uncropped test slices with image dimensions of 96 × 96 pixels are shown in Table 1. The accuracy of identifying slices containing a nasal cavity with the uncropped scans is 96.2 percent with a standard deviation of 2 percent estimated from the 5-fold cross-validation. Out of typically 150 slices per patient intersecting the nasal cavity, this corresponds to only ~6 being misidentified on average. When applied to the cropped scans, the CNN exhibits a slight degradation in performance corresponding to a mean misidentification rate of ~8 slices per patient. Figure 2 (left panel) shows the validation loss and accuracy curves for the slice extraction model. Some example slices rejected and selected by the model are shown in Figure 3.

The results of segmentation using the *ResUNet++* model are shown in Table 2, and an example segmentation is shown in the bottom-right panel of Figure 1. Considering that a Dice coefficient of 1 indicates complete agreement between the predicted and ground truth segmentation masks, the findings reveal the highly accurate identification of nasal cavities by the model. Reliable segmentation is key for the success of the final phase where classification is carried out on these identified areas. Once again, the metrics show that performance degrades slightly when the model is trained and tested on the cropped scans. Figure 2 (middle panel) shows the validation loss and Dice coefficient curves for the segmentation model.

The results of the classification model are shown in Table 3. Classification was applied only to the cropped scans, but training and testing of both the 96 × 96- and 128 × 128-pixel slices were carried out. The ROC-AUC metric shows that very similar performance is obtained between the two image dimensions considered for both the CNN and *ResAttNet* models, but the CNN is consistently more accurate than the *ResAttNet* model. Interestingly, the CNN exhibits a marginally higher accuracy with the higher resolution slices (128 × 128 pixels) in contrast to the *ResAttNet* model, which shows better performance with the lower resolution scans. This is not a statistically significant result given a typical error of 2–3 percent on the metrics found from the 5-fold cross-validation analysis. Figure 2 (right panel) shows the validation loss and Dice coefficient curves for the CNN segmentation model.

**Figure 4:**
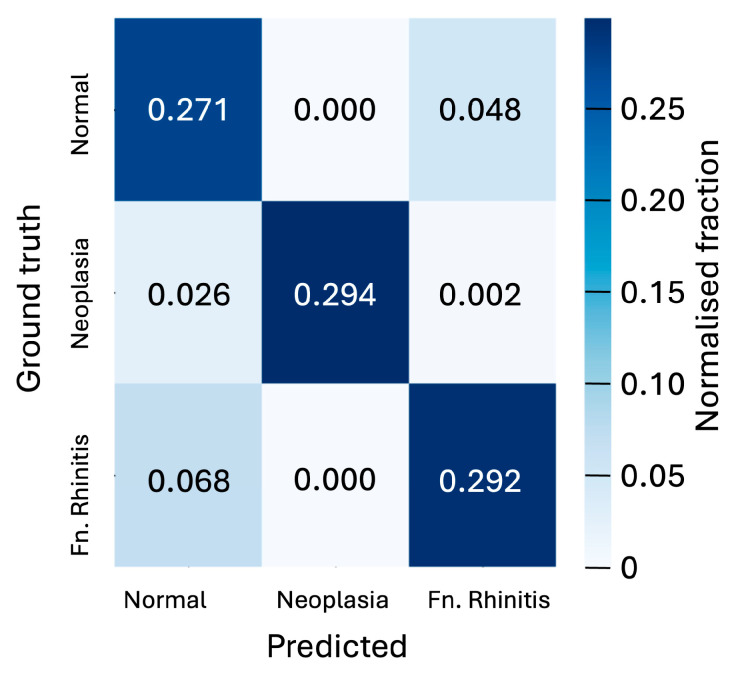
Confusion matrix showing the normalised fraction of the predicted classifications of slices versus the ground truth for the three cases of ‘normal’, ‘neoplasia’, and ‘fungal rhinitis’ considered. Matrix is shown for the 128 × 128-pixel slices classified by the CNN model. The 5-fold cross-validation analysis yields an uncertainty of 0.006 on the fractions displayed in the matrix.

Figure 4 shows the confusion matrix, which breaks down prediction performance by class for the 128 × 128-pixel slices in the case of the CNN model. These results show that the classification accuracy of individual slices is better than 90 percent for neoplasia cases and 84 percent in the absence of pathology, but only 80 percent for rhinitis cases. The model’s strongest confusion stems from the misclassification of rhinitis cases as being pathology-free.

**Figure 5:**
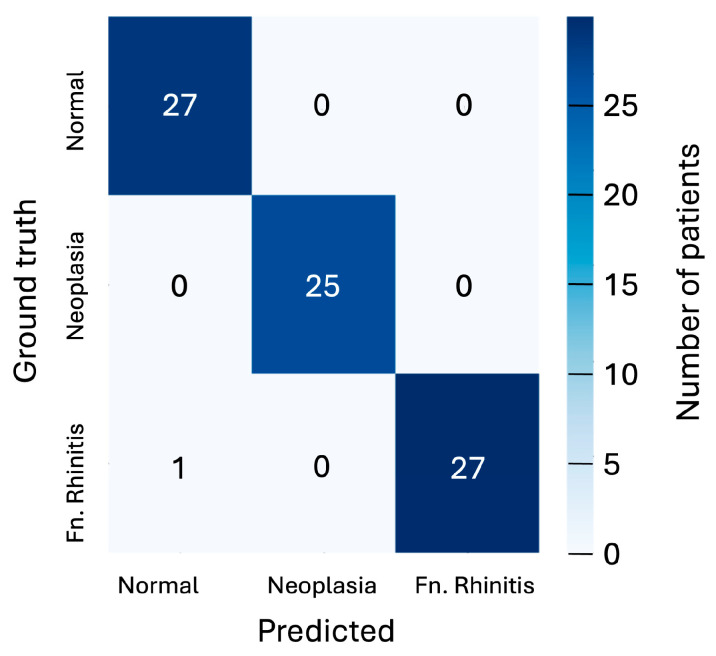
Confusion matrix showing the predicted classifications versus ground truth using aggregated probabilities for each patient for the three cases of ‘normal’, ‘neoplasia’, and ‘fungal rhinitis’ considered. Matrix is shown for the 128 × 128-pixel slices classified by the CNN model.

While a single CNN-classified slice lacks sufficient clinical reliability, the abundance of slices per patient (typically one hundred and fifty, with tens intersecting a pathology if present) allows for a more robust assessment through aggregated probabilities. By selecting the 10 most spatially separated slices within a potential pathology (thus minimising inter-slice correlation), an aggregated probability for each class can be computed by multiplying the individual slice probabilities for that class. The class with the highest aggregated probability is taken as the prediction. Given that the adopted 5-fold cross-validation tests each of the 80 patients, this aggregated probability can be calculated for the entire cohort. The results of this are presented as a confusion matrix in Figure 5, showing that only 1 of the 80 patients is misclassified under this scheme.

## 4. Discussion

This pilot study has demonstrated the efficacy of applying deep learning models to cranial CT imaging data for the diagnosis of nasal disease in dogs. Whilst such technologies have been successfully demonstrated with human scan data [31], the viability of applying such methods to the more varied morphology exhibited in canine scans has remained uncertain. This pilot study demonstrates, with a relatively small volume of training data, that the latest deep learning models can classify canine nasal cavities as belonging to one of the categories defined as ‘normal nasal anatomy’, ‘fungal rhinitis’, and ‘neoplasia’ with a high accuracy of 99%. The work has demonstrated a full end-to-end diagnostic process, starting with the automatic selection of relevant slices from the scan, followed by the identification of the nasal cavities in each scan, and concluding with classification.

The CNN classification model indicates that higher slice image resolution generally correlates with better diagnostic accuracy, though the more complex *ResAttNet* model showed the opposite trend. Due to computational constraints, investigations of higher resolutions were not possible, but it is anticipated that performance would continue to increase with further resolution improvements. Improvement in resolution would require more training data and likely deeper machine learning models, but with this would come the possibility of distinguishing between a greater number of pathology types, including those that show more subtle differences.

A trend observed in this work is that models trained and tested on cropped slices consistently perform worse, albeit only slightly, than those applied to uncropped slices. Since the image dimensions were set equally in both cases, the cropped slices effectively have a higher resolution (i.e., more pixels per physical unit area); this behaviour is therefore likely due to a combination of factors. The model architectures and the number of training slices were kept constant across both cases, suggesting that the reduced performance with cropped slices may be due to the models not being sufficiently deep to capture the complex features within the smaller, cropped regions. Additionally, the reduced amount of data available for training with cropped slices may have hindered the models’ ability to generalise effectively.

A significant limitation of the current study is the relatively small sample size. The use of a relatively small number of CT studies raises the possibility of a skewed dataset, and it is possible that the model was tested on studies in which the differences between subgroups were particularly prominent. In this instance, it would be unclear whether the same performance would be obtained using studies in which the changes were less striking. Similarly, this work considered only three classes (two pathology types). Preliminary tests of the classification model with scans of nonspecific nasal pathologies not seen during training resulted in an inconclusive diagnosis. As an interim step toward training a model capable of handling a significantly wider range of pathologies, further training could include an ‘other’ category. A final, minor limitation of this work is that the three classes considered in this work exhibit a small degree of imbalance, which may introduce bias during model training. However, the aim of the study was not to provide a ready-to-use clinical program but to explore the basic capacity of a machine learning algorithm to distinguish between two of the most common intranasal pathologies in dogs.

Another limitation is that the model developed was only used in medium-to-large dolicocephalic breeds. Whilst such breeds exhibit a larger variation in cranial morphology than humans, the question of how well deep learning methods will perform over a more diverse range of breeds remains unclear. This is a question beyond the immediate remit of the present study, but a brief test was carried out on an additional dataset of scans from small-breed dogs using the models trained on the medium-to-large dolichocephalic breeds. The models continued to perform well over all three phases of slice selection, segmentation, and classification, yielding accuracies consistent with those of the medium-to-large headed breeds. This is most likely attributable to the scale-invariant nature of CNNs, which are at the heart of the models used in this work.

Finally, this study analysed only the pre-contrast bone CT imaging data. Although these more clearly show the nasal cavities and their pathologies upon visualisation of the images, this neglects the additional data contained in the post-contrast bone scans and the pre- and post-contrast soft-tissue scans that are typically obtained. Further studies, preferably including different algorithms, will provide additional independent data to allow for greater classification accuracy if used in combination.

The overall excellent classification results achieved by the current model suggest that machine learning programmes based on CNNs could become a useful tool in accurately diagnosing a varied range of intranasal pathologies in dogs. The next steps towards developing possible routine clinical applications should ideally include a greater range of nasal pathologies (other types of infectious or non-infectious rhinitis alongside neoplastic disease) in a significantly larger cohort of dogs to further test the accuracy of the model in a probable clinical scenario.

## 5. Conclusions

In summary, the results reported in the current study illustrate the possibility of using a deep convolutional neural network for distinguishing between intranasal fungal rhinitis and neoplastic disease in dogs using CT images. Further studies, ideally with larger datasets, are required to determine the learning potential and performance of a CNN in a clinical setting.

## Figures and Tables

**Table 1:** Results of the trained slice extraction CNN applied to cropped and uncropped test scans. Slices with dimensions of 96 × 96 pixels were used.

	Accuracy	F1 Score	ROC-AUC
Uncropped slices	0.962	0.949	0.962
Cropped slices	0.944	0.922	0.941

**Table 2:** Results of the segmentation model applied to cropped and uncropped test scans. Slices with dimensions of 96 × 96 pixels were used. A Dice coefficient of 1 corresponds to perfect overlap between the ground truth mask and the predicted mask.

	Dice Coefficient	F1 Score
Uncropped slices	0.959	0.970
Cropped slices	0.947	0.946

**Table 3:** Performance of the CNN and *ResAttNet* models in classifying individual slices into one of the three categories of ‘normal’, ‘fungal rhinitis’, or ‘neoplasia’. The models were trained and tested on only cropped scans but with both 96 × 96- and 128 × 128-pixel dimensions.

	Accuracy	F1 Score	ROC-AUC
CNN 96 × 96	0.835	0.840	0.939
CNN 128 × 128	0.856	0.858	0.953
ResAttNet 96 × 96	0.821	0.827	0.895
ResAttNet 128 × 128	0.781	0.796	0.873

## Data Availability

The data presented in this study are not publicly available due to privacy and security protection.
